# Effects of different training characteristics in combined resistance and cognitive training on motor and cognitive performance in older adults: A systematic review

**DOI:** 10.1186/s11556-026-00403-3

**Published:** 2026-01-26

**Authors:** Deniz Aminirakan, Dagmar Linnhoff, Bettina Wollesen

**Affiliations:** 1 Institute of Human Movement Science, Universität Hamburg, Hamburg, Germany; 2 Institute of Movement Therapy and movement-oriented Prevention and Rehabilitation, German Sport University Cologne, Cologne, Germany

**Keywords:** Aging, Combined intervention, Dual-task (DT) cognitive training, Cognitive-motor intervention, Strength training, Resistance training

## Abstract

**Background:**

Resistance training as well as cognitive training have shown benefits in improving muscle strength, mobility, and cognitive performance in older adults. However, the optimal strategies for combining these interventions remain unclear. This systematic review evaluates whether combined resistance and cognitive training improve muscle strength, mobility, and cognitive performance in older adults (≥ 65 years) with a specific focus on the most effective training characteristics.

**Methods:**

Following PRISMA guidelines, a systematic search of MEDLINE, Web of Science, and PsycINFO identified Randomized Controlled Trials (RCTs), Controlled Clinical Trials (CCTs), and parallel group trials. Inclusion criteria were: (1) community-dwelling adults aged ≥ 65 without medical conditions, (2) interventions including combined resistance and cognitive training, (3) group comparisons, and (4) reporting of at least one cognitive and one physical outcome. No meta-analysis was conducted; instead, findings were synthesized narratively based on the quality and outcomes of the included studies, using a modified version of the Cochrane tool.

**Results:**

A total of 4682 studies were retrieved, of which nine studies could be included. The number of participants in each group ranged from 18 to 376, with participants distributed across at least two and a maximum of four groups (age range from 65.5 ± 6.3 to 83.9 ± 6.3 years). Three studies used a sequential intervention, while six trials conducted simultaneous Cognitive-Motor Exercise (CME). The studies spanned durations from four to 52 weeks, incorporating various intervention designs and training protocols. Results indicated that programs delivered 2–3 times per week at least 30 min, at moderate- high intensity, for ≥ 12 weeks, most consistently improved both motor and cognitive outcomes.

**Conclusion:**

Combined resistance and cognitive training improved cognitive and motor performance in older adults. Longer-term interventions (12 to 52 weeks) with training frequencies of two to three times per week, appear more likely to yield robust enhancements in cognitive functions and motor abilities. Future research should refine the optimal combination of resistance and cognitive training, considering individual differences and preferences. Also, training characteristics need to be reported in more detail to increase therapeutic validity. This Systematic Review is registered with PROSPERO (CRD42022337354).

**Supplementary Information:**

The online version contains supplementary material available at 10.1186/s11556-026-00403-3.

**Table:** 

Textbox 1 – Resistance training definition	
• According to Bishop et al. [1] resistance exercise is a form of exercise that requires skeletal muscles to exert force to push or pull against resistance with sufficient effort such that the number of repetitions or duration of contractions is limited due to neuromuscular fatigue. The resistancemay include body weight, elastic bands, free weights, and weight or other mechanical machines, among others.	
• Resistance training covers all load ranges with 6–20+ reps, aiming at strength, hypertrophy, or endurance [2].	
• The terms are often used interchangeably, but differ in intensity, reps, and adaptations: strength training emphasizes maximal strength and efficiency, while resistance training supports broader gains [3]. In exercise therapy, these training interventions also include training with small equipment and resistance bands [4]. Nevertheless, throughout this article the term resistance training will be used.	

## Background

In light of the ongoing demographic changes in societies worldwide, the preservation of motor abilities among older adults, which is crucial for independent living, has gained paramount importance [5]. In advanced age, a prevalent functional decline leads to significant issues encompassing decrements in cognitive function and physical performance and results in reduced emotional well-being [6, 7]. However, lifestyle activities, including walking and lifting objects, require balance, coordination, and performance of various physical tasks [8].

Maintaining physical fitness is essential for an independent lifestyle, especially as older adults often experience reduced muscle strength and a decline in skeletal muscle mass, factors associated with increased fall risk, long-term care needs, and higher mortality rates [9–11]. Lower-body strength, particularly in the hip and leg extensors, is critical for preserving mobility in later life. Losses in muscle mass and strength can lead to reduced activity, greater sedentary behavior, functional limitations, impaired mobility, and even cognitive decline [12]. Preserving muscle mass and strength is therefore vital for sustaining independence in older age [13, 14].

In addition, the capacity for DT, such as walking while performing a cognitive task, also deteriorates with age [15, 16]. Thus, two domains are especially important for older adults aiming to remain independent: (1) physical capability, including muscle strength, and (2) cognitive function [17–19].

A growing body of evidence suggests a correlation between physical activity and cognition, sparking increased interest in the potential application of physical exercise as an intervention to preserve independence and cognitive-motor function among older adults [20]. Physical exercise enhances the efficient utilization of cognitive abilities among older adults, resulting in improved daily task performance [21, 22].

Among the various forms of physical exercise beneficial for older adults, resistance training, which involves the use of weights or resistance bands, has been shown to be effective. These activities not only enhance balance and gait performance but also reduce the incidence of falls [23–25]. Moreover, resistance training yields numerous health benefits, enhancing muscular strength, -endurance, and -function, preventing age-associated muscle loss, improving bone density, and enhancing various aspects of functional performance [26–29].

Supplementary cognitive training has been empirically demonstrated to amplify cognitive competencies, including attention, memory, and Executive Functions (EF) [30]. Furthermore, cognitive training has a positive impact on certain physical function outcomes, notably gait performance [13, 14, 22, 31–34]. However, despite extensive research, the most effective dose-response relationship and potential advantages derived from a combination of resistance training with cognitive training remain subjects of ongoing interest. Therefore, it is crucial to investigate the collective impact of cognitive and resistance training on cognitive functionality and muscular strength, including potential interdependencies.

Recent scholarly investigations reveal the efficacy of combined cognitive and physical training interventions on cognitive and physical outcomes, significantly augmenting cognitive-motor function, daily living activities, and overall quality of life in older adults [30, 32, 35–37]. Furthermore, studies suggest a documented superiority of Cognitive-Motor Exercise (CME) over unidimensional training in enhancing cognitive function [38–40]. In this review, CME refers to Wollesen et al. [41], defined as interventions that combine motor tasks (e.g., walking, balance, strength or coordination exercises) with cognitive tasks (e.g., attention, Working Memory (WM), inhibition control, reaction time) performed simultaneously or sequentially, and thereby engaging both domains (motor and cognitive) concurrently. This closely corresponds to DT approaches, with the difference that DTs does not require both areas to be included (e.g., motor-motor or cognitive-cognitive DTs). Performing cognitive and motor tasks simultaneously improves cognitive-motor integration [42, 43], leading to a reduction in fall incidents and bolstering mobility in older adults [44, 45]. Recent research strongly suggests that, in comparison to other forms of physical activity, such as aerobic exercises, resistance training offers a striking enhancement of cognitive benefits for elderly individuals [36, 46–48].

Engaging in CMEs enables older adults to stimulate neuroplasticity, enhancing cognitive resilience and overall brain health [49]. The ‘guided plasticity facilitation’ hypothesis posits that physical and cognitive activities, when performed simultaneously, produce synergistic benefits that exceed the sum of individual gains in cognitive performance [36]. In this interplay of physical and cognitive exertion, mechanisms of attention are refined, and multitasking abilities are strengthened, ultimately contributing to improved cognitive control [50–54]. A recent review by Esmaeilzadeh and colleagues suggested combining exergames with resistance training as a cognitive-motor intervention [55] underpinning the potential spectrum of future interventions for older individuals and the need for additional exploration.

A robust investigation into the contributions from various training types within these combined cognitive-motor interventions would offer valuable insights into the potential reciprocal enhancement of intervention components. However, many of these reviews have either focused broadly on aerobic or multicomponent interventions or emphasized balance and coordination tasks, with less attention to resistance training as the primary physical component [56, 57]. In particular, the dose-response relationships, training progression principles, and interaction effects specific to resistance-based cognitive-motor interventions remain underexplored [11]. This improved understanding could subsequently lead to the creation of inclusive, tailored exercise programs targeting the multifaceted aspects of the aging process, both physical and cognitive.

Therefore, this systematic review aims to investigate the impact of combined resistance and cognitive training on motor and cognitive performance within healthy older adults aged 65 and above. Additional objectives include assessing the efficacy, feasibility, and guidance regarding training loads in the context of the combination of resistance and cognitive training. Key factors such as the frequency, intensity, and duration are considered to identify most effective training characteristics. The main research questions are:

(1) What is the most effective combination of resistance and cognitive training to improve cognitive and motor performance of older adults (≥ 65 years)?
(2) Which recommendations for training load (frequency, intensity, duration, and type) can be synthesized?

## Methods

This systematic review was written following the Preferred Reporting Items for Systematic Review PRISMA 2020 reporting guideline [58]. The protocol was prospectively registered with the International Prospective Register of Systematic Review (PROSPERO) with the registration number (CRD42022337354).

### Eligibility criteria

The following inclusion criteria were employed: (1) healthy older adults with a minimum age of 65 years (or sample mean of ≥ 65 if no minimum age of each participant was stated) who were living independently without a medical condition (2) interventions that include resistance training combined with cognitive training, delivered either sequentially or simultaneously also in multimodal applications (3) at least one comparison group (4) at least one physical (gait or balance performance) and one cognitive outcome or a DT test situation.

The integration of the studies was limited to RCTs, CCTs, or quasi-experimental intervention studies. Studies including (1) persons with an age under 65, (2) brain injuries or cognitive impairments, (3) physical impairments (e.g., using a cane or walker), (4) chronic diseases (e.g., multiple sclerosis or Parkinson’s disease) and (5) secondary analyses of previously reported results were excluded.

### Information sources

We performed a systematic literature search (last update 22 December 2025). The three relevant electronic databases Medline, PsycINFO, and Web of Science (from 1997 to 2025), were searched for studies investigating the effect of combined cognitive and resistance training on mobility and cognitive performance of older adults.

### Search strategy

Searching for the relevant studies was conducted using the established PICO- Principle. According to the PICO scheme we used the following keywords for the literature research: (1) for the population: “elder”, “aging”, “age”, “older adults”, “senior”, “geriatric”; (2) for the intervention: “cognitive training”, “cognitive exercise” “combined cognitive training”, “cognitive training intervention”, “motor cognitive training”, “resistance training”, “resistance exercise”, “strength training”, “motor training” “combined resistance and cognitive training”, “weight training”, “free weights”, and “weight machines”; (3) for the study type: “controlled”, “active Control Groups (CG)”, “CG”, “inactive CG”, “RCT”; and (4) for the outcome: “muscle strength”, “mobility”, “motor”, “cognitive function”, “cognitive performance”, “DT”, “coupled task”, “secondary task”, “strength”.

The search was further limited via database-specific filters to being available in the English language, including human participants, and being articles published in peer-reviewed journals (see the updated search strategy in Supplementary Materials, Tables 4 and 5).

### Selection process

**Fig. 1:**
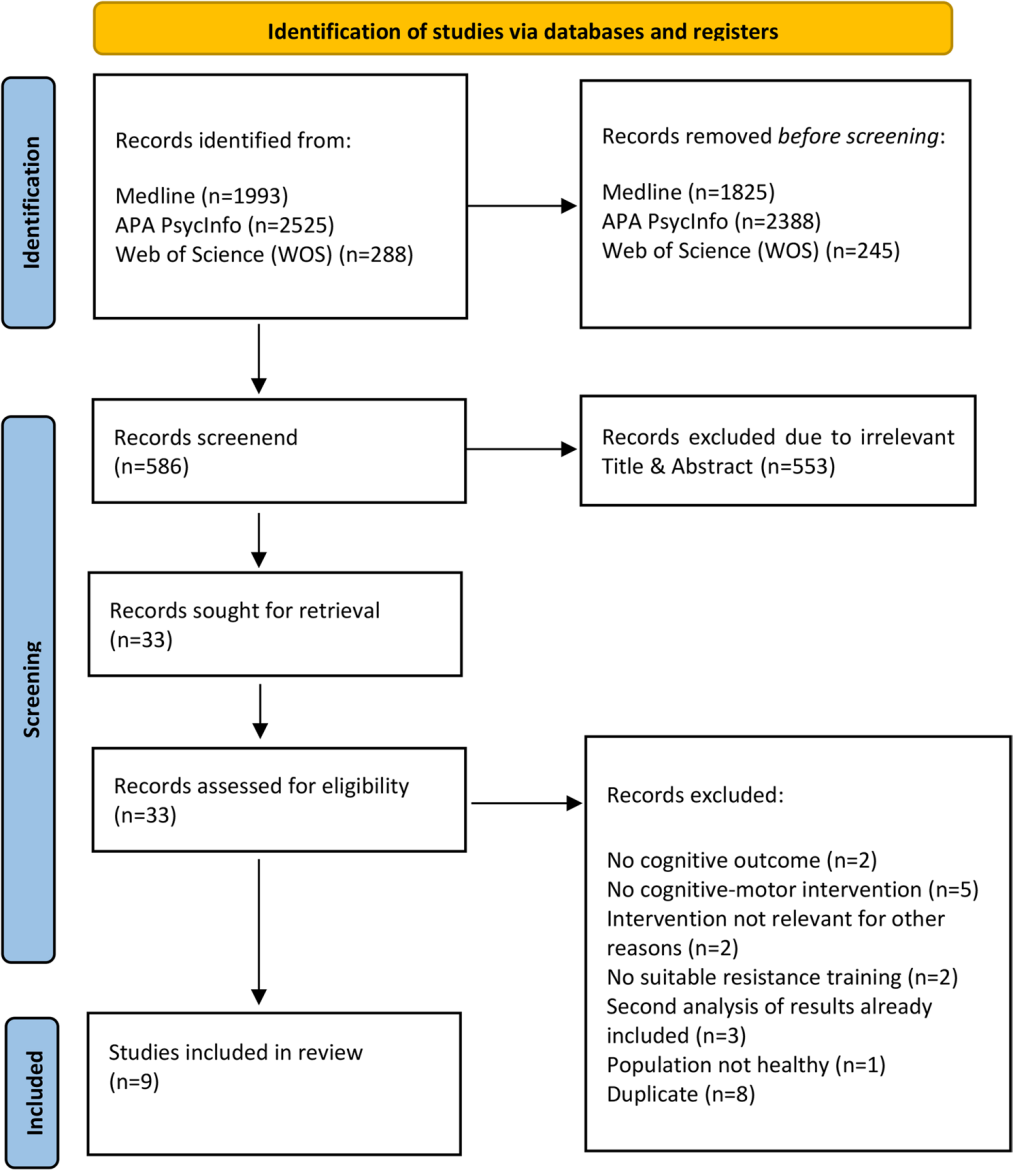
PRISMA flow chart for the steps of the literature searching and screening process [62]

 The initial screening process was performed by two researchers (DA and DL) and involved analyzing the title and abstract of the studies to identify and exclude thematically non-eligible studies following PRISMA methodology [58] (Fig. 1).

After this first screening stage, all remaining articles were integrated into the full-text screening. The full-text screening was performed by two researchers independently (DA and DL), and any inconsistencies were resolved through discussion with all of the authors (DA, DL, and BW).

### Data collection process

Data extraction of the remaining articles was done by one author (DA) and controlled by a second author (DL). The extracted study characteristics included general characteristics (year of publication, population characteristics including age and gender, and study designs, the characteristics of the resistance training (e.g. type and involved muscles, loading and volume, frequency, progression, and adaptation) as well as of the cognitive training (e.g. type, tasks, addressed domains, time, progression and adaptation of tasks)). The extraction of information followed the recommendations of Hecksteden et al. [59].

We categorized the cognitive outcomes into global cognition, processing speed, attentional control, EF, and memory according to test specifications provided in the manuscripts. To categorize the physical outcomes, we separated strength performance outcomes (e.g., Sit to Stand Test (STS), handgrip strength) from locomotor outcomes that reflect daily activity and mobility (e.g., walking speed, Timed Up and Go Test (TUG)). The details are reported in Table (1).

**Table 1:** Characteristics of included studies

General				Outcomes	
Authors (Year)	Study aim	Population *N* (total); *N* (analysed; gender); Groups *n* (Mean age ± SD)	Training Type	Cognitive Outcome Measures	Motor Outcome Measures
Jardim et al. (2021)	Tested the effects of dual-task multimodal physical exercise training and cognitive stimulation on cognitive and physical function in healthy older adults.	*N* = 91 (total); *N* = 72 (analysed; 84% f); IG: *n* = 41 (67.39 ± 0.90); pCG: *n* = 31 (67.87 ± 0.99)	Simultaneous	Global cognition *Assessment*: - Mini-Mental-Status-Test (MMSE) Processing Speed n.a. Attentional control *Assessment*: - Rapid visual information processing-Test (RVP) by CANTAB Executive function *Assessment*: - Paired associates learning test (PAL) - PAL Stages completed Score (SC)/PAL Number of patterns succeeded Score (NPS)/ PAL Total Trials Adjusted Score (TTA) - CERAD Word List Memory	Strength Lower limb strength *Assessment*: - Chair rise test Mobility Gait speed, Functional mobility *Assessment*: - 6-Minute walking test (6MWT) - Timed up and go (TUG) Balance n.a. Cognitive-motor performance *Assessment*: - Walking while talking test − 6-minute DT-walking speed
Nishiguchi et al. (2015)	Tested the effects of a physical-cognitive exercise program on cognitive function and brain activation efficiency in older adults.	*N* = 48 (total); *N* = 48 (analysed, 45,8% f); IG: *n* = 24 (73.0 ± 4.8); pCG: *n* = 24 (73.5 ± 5.6)	Simultaneous	Global cognition *Assessment*: - Mini-Mental-Status-Test (MMSE) Processing Speed *Assessment*: - Simple Reaction Time Attentional control n.a. Executive function *Assessment*: - ∆ TMTA-B - N-back test - Wechsler memory scale revised (WMS-R - logical memory) immediate and delayed	Strength Lower limb strength *Assessment*: - Chair rise test (5 times) Mobility Gait speed, functional mobility *Assessment*: -10-meter walking test - TUG Balance n.a. Cognitive-motor performance n.a.
Norouzi et al. (2019)	Comparing a motor-cognitive dual-task training (mCdtt) vs. a motor-motor dual-task training (mMdtt) in older adults’ population.	*N* = 60 (total); *N* = 60 (analysed; 100% m) IG (mCdtt) *n* = 20 (68.51 ± 3.65); aCG (mMdtt): *n* = 20 (68.31 ± 4.2); pCG: *n* = 20 (68.10 ± 3.71)	Simultaneous	Global cognition *Assessment*: - MMSE Processing Speed n.a. Attentional control n.a. Executive function *Assessment*: - N-back test	Strength n.a. Mobility n.a. Balance *Assessment*: - Berg Balance Test Cognitive-motor performance n.a.
Salazar-Gonzalez et al. (2015)	comparing the effects of a combined physical cognitive exercise intervention on DT-gait in older adults.	*N* = 376 (total); *N* = 286 (analysed; 82,5% f); IG: *n* = 143 (71 ± 3.31); pCG: *n* = 143 (74 ± 6.31)	Simultaneous	Global cognition *Assessment*: - MMSE Processing speed n.a. Attentional control n.a. Executive function n.a.	Strength n.a. Mobility n.a. Balance n.a. Cognitive-motor performance *Assessment*: - DT walking performance (speed, step length, width, cadence, double support) counting backwards) - DT cognitive task performance (Number of Digits/animals)
Laatar et al. (2018)	Comparing simultaneous physical-cognitive training vs. only physical training on postural performance in older adults.	*N* = 30 (total); *N* = 24 (analysed gender not reported); IG(PCG): *n* = 12 (66.29 ± 3.61); aCG(PG): *n* = 12 (67.45 ± 2.38)	Simultaneous	Global cognition n.a. Processing Speed Reaction time *Assessment*: - simple reaction time test (SRT) Attentional control n.a. Executive function n.a.	Strength Lower limb strength *Assessment*: - Chair rise test Mobility Gait speed, Functional mobility *Assessment*: - 10-meter walking test - TUG Balance Static balance, dynamic balance *Assessment*: - COP- sway while quiet standing - COP- sway while functional reach test (FR) (COP- sway tested on a static stabilometric platform) Cognitive-motor performance DT gait speed DT balance *Assessment*: -DT Walking while conversing on a phone - DT postural balance on a platform with a button press task
Uemura et al. (2012)	Evaluating a specific exercise intervention Dual- task switch exercise (DSE) to improve gait initiation for fall prevention in the elderly population	*N* = 18 (total); *N* = 15 (analysed; 79.4% f); IG (DSE): *n* = 8 (82.4 ± 5.9); aCG: *n* = 7 (82.4 ± 6.8)	Simultaneous	Global cognition n.a. Processing Speed Reaction time *Assessment* - reaction time in gait initiation test Attentional control n.a. Executive function n.a.	Strength n.a. Mobility Gait speed, gait initiation *Assessment*: −10-meter walking test - COP displacement during gait initiation Balance n.a. Cognitive-motor performance *Assessment*: (All measures performed while counting backwards) - DT COP displacement during gait initiation - DT steady-state gait speed - DT reaction time
Desjardins-Crépeau et al. (2016)	Comparing combined physical and cognitive interventions on physical fitness and neuropsychological performance in healthy older adults.	*N* = 125 (total); *N* = 76 (analysed; 69,7% f) IG: *n* = 20, (73.2 ± 6.3); aCG AR 1: *n* = 22, (72.7 ± 7.4); aCG ST 2: *n* = 16, (70.9 ± 7.4; aCG CL 3: *n* = 18, (72.5 ± 7.0)	Sequential	Global cognition n.a. Processing Speed n.a. Attentional control *Assessment*: - Baddeley-single task test (BST) - Baddeley-Dual task test (BDT) Executive function *Assessment*: - Color-word interference test (inhibition) - Color-word interference test (switching) -TMT(A-B) - Rey Auditory Verbal Learning Test (RAVLT) - Total 5 - Immediate recall - Delayed recall	Strength Handgrip strength Lower limb strength *Assessment*: - Hand-dynamometer - Chair rise test Mobility Gait speed, functional mobility *Assessment*: -6MWT - TUG - Modified physical performance test (PPT) Balance n.a. Cognitive-motor performance n.a.
Sipilä et al. (2021)	Comparing physical and cognitive training (PTCT) with physical training (PT) alone among 70-to-85-year-old men and women.	*N* = 314 (total); *N* = 314 (analySed; 60% f); IG(PTCT): *n* = 155 (74.4 ± 3.9); aCG(PT) *n* = 159 (74.5 ± 3.7)	Sequential	Global cognition n.a. Processing Speed n.a Attentional control n.a. Executive function *Assessment*: - Stroop Color-Word Test - ∆ TMT (A-B) - Verbal Fluency Test - CERAD Word List Memory	Strength Handgrip strength, Lower limb strength *Assessment*: - Hand-dynamometer - Isometric knee extension force Mobility Gait speed, normal walking, physical performance *Assessment*: -10-meter walking test - 6-minute walking distance - SPPB Balance: n.a. Cognitive-motor performance Dual-task gait speed *Assessment*: - 6-minute walking distance while doing a visuospatial task
Van het Reve et al. (2014)	Comparing cognitive-motor training vs. motor training alone: exploring the additional effect of the supplemented cognitive training	*N* = 182 (total); *N =* 145 (analysed; 69.7% f); IG(SBC): *n* = 69 (81.1 ± 8.3); aCG (SB): *n* = 76 (81.9 ± 6.3)	Sequential	Global cognition *Assessment*: Mini-Mental-Status-Test (MMSE) Processing Speed Psychomotor speed (simple reaction time) *Assessment*: - Button press task Attentional control Divided attention *Assessment*: - Vienna Test System Executive function *Assessment*: - TMT (A-B)	Strength n.a. Mobility Functional mobility *Assessment*: - SPPB - Expand timed up and go test (ETGUG) - ETGUG (gait initiation) Balance n.a. Cognitive-motor performance Dual-task cost of walking (velocity, step time, and -length) *Assessment*: (all measures performed while subtracting numbers or naming animals) - DT walking performance at preferred speed - DT walking performance at fast speed

Legend: *aCG*  active control group, *CERAD*  Consortium to establish a registry for Alzheimer’s disease, *COP*  center of pressure, *DT*  dual task, *IG*  intervention group, *n.a*. not available, *PG*  physical Group, *PCG*  physical cognitive group, *pCG*  passive control group, *SPPB*  Short Physical Performance Battery, *TMT*  trail making test, *SB * strength-balance, *SBC* Strength-balance-cognitive

### Study risk of bias assessment

For the quality assessment, an adaptation of the modified criteria by Van Tulder et al. (2003) [60] was used. In addition to the original criteria proposed by Van Tulder et al. [60], we added three additional criteria as proposed by Wollesen et al. [61]: appropriate description of the intervention (intensity and duration), appropriate description of the measurements (for both cognitive and motor domains), and adequate report of measurement results and statistical analysis. Ending up in nine full plus three additional categories, making up a possible score of 12 points as the highest quality score. Studies scoring 9–12 were considered high quality, 5–8 as fair quality, and 0–4 as low quality.

The quality assessment was conducted independently by two authors (DA and DL). After separately rating the study quality with this modified tool, the scores were compared for each category, and in case of inconsistency, a third author (BW) was consulted for the final vote. The results of the risk of bias assessment are summarized in Table (2).

**Table 2:** Risk of bias and quality assessment based on 12-Point Quality Scoring System, 9-12 high quality, 5-8 fair quality, 0-4 low quality

Study	a*	b*	c*	d*	e-m (*)	e-c (*)	f*	g*	h*	i*	j-m	j-c	k-m	k-c	l	Score of 12	Orig. Score*	General remarks
Desjardins-Crépeau et al. (2016)	x	x	(x)	x	-	x	x	x	x	-	u	x	x	x	x	10	8.5	e-m: the training was mixed with little resistance part
Jardim et al. (2021)	-	-	u	u	-	x	x	(x)	u	-	x	x	x	x	x	4	1.5	e-m: the training was mixed and consisted of 50% resistance
Laatar et al. (2018)	x	u	x	u	-	-	u	x	u	-	-	-	x	-	x	4.5	3	e-m/ e-c: the motor training in CG contains cognitive components and motor training is mixed k-c: the cognitive outcome is simple reaction time with no rationale provided
Nishiguchi et al. (2015)	x	x	x	x	-	x	x	x	u	-	x	-	x	x	(x)	8	6.5	e-m: < 50% only strength most of the motor training seems aerobic (steps and stepping tasks); j-c: no information on targeted domains and progression of the cognitive portion of training
Norouzi. et al. (2019)	x	u	x	u	x	x	u	x	u	-	(x)	(x)	(x)	x	x	5.5	4	
Salazar-Gonzalez et al. (2015)	-	x	-	u	x	x	(x)	(x)	u	-	-	-	(x)	-	x	3	2	j-m: no information about targeted muscle groups and intensity adaptation j-c: no targeted dimensions k-c: outcome and training are similar (maybe same) tasks, unclear whether it was controlled for priorization
Sipilä et al. (2020)	x	x	x	x	-	x	(x)	x	u	-	x	x	x	x	(x)	7.5	5.5	e-m: mixed training intervention with < 50% motor training
Uemura et al. (2012)	x	u	(x)	u	-	-	u	x	u	-	-	(x)	(x)	(x)	x	3	2	e-m/ e-c: the additional training in both groups contains different cognitive components and motor training is mixed j-m: no intensity and progression for the resistance part
van het Reve et al. (2014)	x	u	x	x	x	x	x	x	u	x	x	x	x	x	x	10	7	

Risk of bias and quality assessment based on 12-Point Quality Scoring System, 9–12 high quality, 5–8 fair quality, 0–4 low quality

a – acceptable method of randomization; b – concealed treatment allocation; c – similar group values at baseline; d – blinded assessor; e-m – avoided or similar cointerventions (motor training); e-c – avoided or similar cointerventions (cognitive training); f – acceptable compliance; g – acceptable dropout rate; h – similar timing of the outcome assessment in all groups; i – intention-to-treat analysis; j-m – appropriate description of the motor intervention (content, intensity, duration); j-c – appropriate description of the cognitive intervention (content, frequency, exact description of time spent within the activity); k-m – appropriate description of the motor measurements; k-c – appropriate description of the cognitive measurements; L – adequate report of measurement results and statistics. x – “yes” score. “-” – “no” score. (x)- undertaken with general remarks. “u” unclear

## Results

The flow chart of the study selection process is illustrated in Fig. 1. In summary 33 articles underwent full-text screening for eligibility.

Common reasons for exclusion included the absence of either cognitive outcomes [63] or motor outcomes [64], or the inclusion of non-healthy participants [65]. Duplicates were also removed during this stage, resulting in the inclusion of nine studies in the systematic review.

### Study characteristics

Overall, a total of *N* = 1,198 participants were reported, consisting of female (*n* = 802) and male (*n* = 366) participants. While information on gender distribution was not always available [66], (see Table 1) most studies included both male and female participants. The study sample sizes ranged from 18 to 376 participants, with participants distributed across at least two and up to four groups per study. The mean age of participants across the studies ranged from 65.5 ± 6.3 to 83.9 ± 6.3 years.

The nature of the CG differed between the studies and comprised passive CG with no alternative intervention [67–69] or active CG (alternative training, primarily physical-only training) [66, 70–73]. Both passive and active CGs were used by Norouzi et al. [74]. The duration of interventions varied considerably, ranging from 4 weeks [74] to 52 weeks [72]. The most common intervention duration was 12 weeks, reported in five studies (see Table 1).

### Intervention details

The characteristics of the interventions, including the type of intervention, descriptions of motor and cognitive tasks, and details about frequency, session duration, and Total intervention Time, are summarized in Table 3.

**Table 3:** Data extraction of included studies, assessment timelines, total time involved, progression methodologies, and intensity levels, for both motor and cognitive performance

Authors (Year)	Description of Cognitive Training	Cognitive Training Time, Duration and Frequency, total time	Cognitive Training Progression	Description of Resistance Training	Resistane Training Time, Duration and Frequency, total time	Resistance Training Intensity	Resistance Training Progression	Results Cognitive performance	Results Motor performance
Simultainious Trainng and passive Control Group									
Jardim et al. (2021)	MS : Sensory responses to visual and auditory stimuli.	12 wk, 75 min ; 2x p.w ; tt : 1800 min	increasing difficulty of the tasks throughout the sessions	Resistance training prioritized multiarticular and global exercises (squat and bench press)	12 wk, 30 min ; 2x p. w ; tt : 720 min	60–70% MHR	According to ACSM recommendations: increasing number of repetitions or increasing load every 2 week	Global cognition n.a. Processing Speed n.a. Attentional control *RVP* IG: ↑ CG: n.s. Executive function *PAL SC Score* IG: ↑ CG: n.s. *PAL NP Score* IG:↑ CG: n.s. *PAL TTA Score* IG:↑ CG: n.s. *CERAD Word List Memory* Immediate IG:↑ CG:↑ Evocation IG:↑ CG: n.s. Regcognition IG: ↑ CG: n.s.	Strength Lower limb strength *Chair rise test* IG:↑ CG: n.s. Mobility Gait speed *6MWT* IG: ↑ CG: n.s. Functional mobility *TUG* IG:↑ CG: n.s. Balance n.a. Cognitive-motor performance *DT walking speed* IG: ↑ CG: n.s.
Nishiguchi et al. (2015)	MC DT Exercises in 3 Categories: Verbal fluency during stepping, numerical body part coordination, spatial awareness.	12 wk, 60 min; 1x p. w; tt: 720 min	Intensity and difficulty of the three exercises were gradually increased over the 12 weeks	progressive muscle resistance training (no further information)	12 wks, 15 min; 1x p.w; tt: 180 min	Based on recommendations from ACSM and AHA	n.a.	Global cognition *MMSE* IG: n.s. CG : n.s. Processing Speed *SRT* IG:↑ CG: ↑ Attentional control n.a Executive function *∆ TMT (A-B)* IG:↑ CG: n.s. *N-back test* IG: n.s. CG: n.s. *(WMS-R I + II)* IG: ↑ CG: n.s.	Strength Lower limb strength *Chair rise test* IG:↑ CG: n.s. Mobility Gait speed *10-meter walking test* IG: ↑ CG: n.s. Functional mobility *TUG* IG/CG: n.s. Balance n.a. Cognitive-motor performance n.a.
Norouzi et al. (2019)	IG1: DT paradigm: (e.g. throwing a ball up and down; throwing a bag; holding a bag; balancing the cup on the palm; holding medicine ball in both hand) IG2:Different WM-specific tasks: (e.g. backward number counting; mental arithmetic); spelling; backward counting; remembering words, images, colors, and shapes	IG1/IG2: 4 wk, 60–80 min 3 x p. w; tt: 840 min	n.a.	IG1/ IG2: resistance exercise (isokinetic)	IG1/IG2: 4 wk,60–80 min; 3 x p.w; tt: 840 min	Speed in degrees per second	Reported in degrees per second for the isokinetic machines	Global cognition n.a. Processing Speed n.a. Attentional control n.a. Executive function *N-back test* IG1 (motor-motor DT) ↑ IG2 (cognitive-motor DT) ↑sig.improvement CG: n.s.	Strength n.a. Mobility n.a. Balance *Berg Balance Test* IG1 (motor-motor DT) ↑ IG 2 (cognitive-motor DT) ↑ CG: n.s. Cognitive-motor performance n.a.
Salazar-Gonzalez et al. (2015)	DT -walking (naming days of the week, months, and seasons); zig-zag walking, hurdles, ball passing, walking while listening.	12 wk, 20 min; 3x p.w; tt: 720 min	n.a.	resistance training with dumbbells and leggings, starting with 227 g	12 wk, 20 min; 3x p.w; tt: 720 min	n.a.	increasing the weight until the participant could tolerate 680 g	Global cognition n.a. Processing Speed n.a. Attentional control n.a. Executive function n.a.	Strength n.a. Mobility n.a Balance n.a. Cognitive-motor performance *DT gait performance* IG:↑ CG: n.s. *DT Cognitive task performance* (Number of Digits/animals) IG/CG: n.s. group effects.
Simultainious Trainng and active Control Group									
Laatar et al. (2018)	different WM-specific tasks: (e.g. Counting, calculations, visual searching, naming things, remembering things, description, visual imagination)	24 wk, 30 min; 3 x p. w; tt: 1080 min	Progressive interventions encompassed balance, strength, and sensory exercises over time.	IG: Standing on toes, body weight squat, side leg, sit to standing up from a chair, hip flexion, abdominal exercises, back exercises aCG1: Balance exercise Semi-tandem, tandem stand, on one leg, heel to toe walking, knee marching, star-excursion, backward/sideward walking, head rotating, making circles with head Combined balance-resistance Standing on toes, body weight squat, side leg, sit to standing up from a chair, hip flexion, abdominal exercises, back exercises	IG:24 wk, 30 min; 3 x p.w; tt: 1080 min aCG1: 24 wk, 30 min; 3 x p.w; tt: 1080 min	n.a.	Progressive interventions encompassed balance, resistance, and sensory exercises over time.	Global cognition n.a. Processing Speed *SRT* IG:↑ aCG1:↑ Attentional control n.a. Executive function n.a.	Strength Lower limb strength *Chair rise test* IG:↑ aCG1:↑ Mobility Gait speed *10-meter walking test* IG:↑ (post and follow up) aCG1:↑ (post, not follow up) Functional mobility *TUG* IG:↑ aCG1:↑ Balance Static balance *COP - sway in quiet standing* IG:↑ aCG1:↑ Dynamic balance *COP - sway in functional reach test* IG:↑ aCG1:↑ Cognitive-motor performance DT gait speed *Walking while conversing on a phone* IG:↑ aCG1: n.s. DT balance *Postural balance while button press task* IG:↑ aCG1: n.s.
Uemura et al. (2012)	IG: Weeks 7–12: Forward counting and Japanese alphabet recitation. Weeks 13–24: Naming animals, vegetables, or professions added. (performed more complex cognitive tasks over time) aCG1:Weeks 7–12: Forward counting and Japanese alphabet recitation. Weeks 13–24: Naming animals, vegetables, or professions added. (performed cognitive tasks but at a steady pace)performed cognitive tasks but at a steady pace	IG: 18 wk, 5*** min; 1 x p.w; tt: 150 min aCG1: 18 wk, 5*** min; 1 x p.w; tt: 150 min	In both groups, the cognitive load was progressively increased during 24 wk program	IG: seated no equipment exercises: resistance training of lower limb (e.g., knee extension, calf raise, toe raise) aCG1: seated no equipment exercises: resistance training of lower limb (e.g., knee extension, calf raise, toe raise) steady state walking training(10- meter forward, backward, and laterally)	IG: 24wk, < 25 min****; 1 x p.w; tt: <600 min aCG1: 24wk, < 25 min****; 1 x p.w; tt: <600 min	n.a.	n.a.	Global cognition n.a. Processing Speed Reaction time in gait initiation IG: n.s. aCG1: n.s. Attentional control n.a. Executive function n.a.	Strength n.a. Mobility Gait speed *10-meter walking test* IG: n.s. aCG1: n.s. Gait initiation *COP displacement during gait initiation* IG:↑ aCG1:↑ Balance n.a. Cognitive-motor performance *Dual-task COP displacement during gait initiation* IG: n.s. aCG1: n.s. *DT steady-state gait speed* IG:↑ aCG1: n.s. *DT reaction time* IG:↑ aCG1: n.s.
Sequential Training and active Control Group									
Desjardins-Crépeau et al. (2016)	Visual discrimination tasks: Numbers and shapes in single pure, single mixed, and dual mixed (DT ) formats with speed and accuracy feedback. CG CL3: computer and diverse software(e.g., Word &Excel),initiation to Internet(e.g., searching engines, web sites, online games, etc.)	IG: 12 wk, 60 min; 1 x p. w; tt: 720 min CG CL3: n.a.	n.a.	IG: resistance cables; Lower body training targeting quads, hamstrings, hip muscles, and ankle flexors. CG AR1: cardiorespiratory fitness on treadmile /lower body muscle strength, targeting quads, hamstrings, hip muscles, and ankle flexors CG ST2: stretching exercise targeted entire body	IG: 12 wk, 15 min; 3 x p.w; tt: 540 min CG AR1: 12 wk,30-min cardiovascular training; 3 x p.w; tt: 1080/ CG ST2: 12 wk, 50 min; 3 x p.w; tt: 1800 min	IG: n.a. CG AR1: modrate to high intensity borg scale, CG ST2: n.a.	n.a.	Global cognition n.a. Processing Speed n.a. Attentional control *BDT ST* IG:↑ CG AR 1:↑ CG ST 2:↑ CG CL 3:↑ *BDT DT* IG: n.s. CG AR 1: n.s CG ST 2: n.s CG CL 3: n.s. Executive function *CWIT inhibition* IG:↑ CG AR 1:↑ CG ST 2:↑ CG CL 3:↑ *CWIT switching* IG:↑ CG AR 1: n.s CG ST 2: n.s CG CL 3: n.s. *TMT A* IG:↑ CG AR 1:↑ CG ST 2:↑ CG CL 3:↑ *TMT B* IG: n.s. CG AR 1: n.s CG ST 2: n.s CG CL 3: ↑ *RAVLT - all test conditions* IG: n.s. CG AR 1: n.s CG ST 2: n.s CG CL 3: n.s.	Strength Hand grip strength *Hand-dynamometer* IG: n.s. CG AR 1:↑ CG ST 2: n.s CG CL 3: n.s. Lower limb strength *Chair rise test* IG:↑ CG AR 1:↑ CG ST 2:↑ CG CL 3:↑ Mobility Gait speed *6MWT* IG:↑ CG AR 1:↑ CG ST 2:↑ CG CL 3:↑ Functional mobility *TUG* IG: n.s. CG AR 1: n.s CG ST 2: n.s CG CL 3: n.s. *PPT* IG:↑ CG AR 1:↑ CG ST 2:↑ CG CL 3:↑ Balance n.a. Cognitive-motor performance n.a.
Sipilä et al. (2021)	iPASS cognitive training: letter updating, predictability.	52 wk; 15–25 min; 3–4 x p. w; tt: 2340–5200 m	Adjusted individually as a function of prior performance	IG: From week 8–9: resistance exercises for the lower body, trunk, and upper body muscles + home based exercises for the lower body with resistance bands aCG1: resistance and balance training, walking and balance training and home exercises and self-guided moderate activity resistance training: gym equipped with machines + walking and balance training: 400 M outdoor circulsr walking lane, indoors 200-meter oval track with dynamic balance + home based exercises for the lower body with resistance bands/aerobic activity	IG:52 wk, 30 min; 2–4 x p.w ; tt: 3120–6240 min aCG1: Resistance/balance 52 wk,45 min;1 x p.w; tt: 2340 min walk /balance 52 wk,15 min; 1 x p.w; tt: 780 min Home exercise: 52 wk,20–30 min;2–3 x p.w; tt: 2080–4680 min/ aerobic: 150 min p.w; bouts of 10 min;tt:7800 min aCG1: 52 wk;4–5 x p.w; tt:12,960 − 15,600 min	n.a.	IG: Adjustments were made to the training program based on the specificity, volume, and intensity of the exercises. If the target number of repetitions was surpassed, the resistance level was increased accordingly. aCG1 : Balance reducing hand, base, and vision support resistance Home exercise: Three different resistance- band	Global cognition n.a. Processing speed n.a. Attentional control n.a. Executive function *Stroop Color-Word Test* IG:↑ aCG1:↑ (larger effect for IG) *∆ TMT(A-B)* IG:↑ aCG1:↑ *Verbal fluency* IG: n.s. aCG1: n.s. *CERAD* IG:↑ aCG1:↑	Strength Hand grip strength *Hand-dynamometer* IG: n.s. aCG1: n.s. Lower limb strength *Isometric knee extension* IG: n.s. aCG1: n.s. Mobility Gait speed *10-meter walking test* IG:↑ aCG1:↑ Normal walking *6 min walking distance* IG:↑ aCG1:↑ Physical performance *SPPB* IG: n.s. aCG1: n.s. Balance n.a. Cognitive-motor performance DT gait speed IG:↑ aCG:↑
van het Reve et al. (2014)	CogniPlus Computer training: Real-life scenarios for alertness and attention in driving.	12 wk, 10 min; 3x p.w; tt: 360 min	Automatic adjustment of training intensity based on the abilities of the performer”	IG: resistance training of: hip extensors, ab- and adductors, knee flexors and extensors, ankle dorsi- and plantar flexors, abdominal- and back muscles with machines aCG1: training focus on lower extremity muscle function, resistance combined balance training resistance training hip extensors, ab- and adductors, knee flexors and extensors, ankle dorsi- and plantar flexors, abdominal- and back muscles with machines balance training one leg stand, tandem stand, walking on heels, backward/sideward walking, turns, sit to stand, knee squats	IG: 12 wk, 30 min; 2xp.w; tt: 720 min aCG1: 12 wk, 40 min; 2xp.w; tt: 720 min	IG/aCG: Intensity, progression and duration of the program were based on previously published recommendations	Intensity, progression, and duration of the program were based on previously published recommendations	Global cognition n.a. Processing Speed simple reaction time *Button press task* IG:↑ aCG1:↑ Attentional control *Vienna Test System* IG:↑ aCG1: n.s. Executive function *TMT A* IG:↑ aCG1:↑ *TMT B* IG:↑ aCG1:↑	Strength n.a. Mobility Functional mobility* ETGUG* IG: ↑ aCG1:↑ *ETGUG (gait initiation)* IG:↑ aCG1: n.s. Physical performance *SPPB* IG: ↑ aCG1:↑ Balance n.a. Cognitive-motor performance DT cost of walking *Preferred speed* IG:↑ aCG1:↑ *(Interaction effect only for step length)* IG:↑ aCG1: n.s. *Fast speed (fast speed)* IG:↑ aCG1:↑ *(Interaction effect for step length &variability)* IG:↑ aCG1: n.s.

Legend: *MS* Motor sensory, *MC* Motor cognitive, *wk* Week , *p.w *pro week, *Min* minute , *tt *total time, *MHR* Maximum Heart rate, *RVP* rapid visual Processing, *6MWT* 6 meter walking test, *IG *Intervention goup, *CG* control group, *WM* working memory, ↑= sig. improvement, *Sig* significant, *n.s.* not significant,  *n.a.* not available, PAL Paired associates learning test, PAL* SC* PAL Stages completed, PAL* NPS* Number of patterns succeeded, PAL *TTA* Total Trials Adjusted, *DT* Dual task, *CERAD* Consortium to establish a registry for Alzheimer's disease,* MC DT *motor cognitive dual task, *ACSM* American College of Sports Medicine , *AHA* American Heart Association, *COP* center of pressure, *DT COP* dual task center of pressure, *SPPB* short physical performance, *CWIT* Color word interference test, *AR* Aerobic and resistance, ST stretching & toning, *CL* Computer lesson, *aCG* active control group, *BDT ST* Baddeley Test  single task, *BDT DT* baddeley dual task, *ETGUG* expanded time up and go, *WMS-R* Wechsler memory scale revised, *RAVLT* Rey Auditory Verbal Learning Test *PPT* Physical Performance Test, *VFT* Verbal Fluency Test, *TMT* Trail Making Test, *MMSE* Mental State Examination

Resistance training was typically performed using body weight, free weights, strength bands, dumbbells, or exercise machines, and most frequently targeted the lower body, followed by full-body training. No study exclusively performed resistance training without incorporating other forms of physical exercise (e.g., balance, endurance, or coordination) into the protocol. The proportion of resistance training varied across the exercise programs and was not always described in detail.

Cognitive training, combined with resistance training, was performed *simultaneously* with physical training in six studies [66–70, 74]. In three studies [71–73], cognitive training was conducted separately on different days, using computer-based tasks. The cognitive training generally targeted EF such as inhibitory control, and WM, using tasks like backward counting, naming and memory tasks, searching tasks, and inhibition control tasks.

The duration of intervention sessions ranged from a minimum of 15 min [68] to a maximum of 80 min [74]. Over the study periods, the accumulated intervention time ranged from 150 min to 6240 min, with 150 to 5200 min allocated for cognitive training and 180 to 6240 min for resistance training.

### Simultaneous interventions

The intervention protocols varied significantly across the studies in terms of content (different combinations of cognitive and resistance training exercises) and dose (session durations ranging from 5 min to 80 min for cognitive training, and from 15 min to 80 min for resistance training [66–68, 70].

The most frequently employed intervention protocol involved a combination of resistance and balance training with physical-cognitive exercises. In these protocols, cognitive tasks (e.g., WM tasks, verbal fluency tasks, and visual search tasks) were integrated into physical activities like walking, dancing, or stepping [66–70]. One study [74] incorporated isokinetic machines for resistance exercises while simultaneously performing cognitive tasks as part of a DT protocol.

### Sequential interventions

The second most commonly used intervention protocol involved resistance training either on machines or with resistance bands, combined with computerized cognitive training conducted on separate days [71–73].

The duration of training sessions, which included all types of interventions, ranged from 30 to 90 min per session, with 15 to 30 min specifically allocated to resistance training and 10 to 60 min allocated to cognitive training. In one study, participants engaged in computerized cognitive training for 10 min, 3 times per week, and physical exercise for 30 min, 2 times per week [73]. The other two studies implemented protocols combining 15 min of cognitive training with resistance exercises [71] or 15–25 min of cognitive tasks with 30 min of resistance training [72].

### Quality assessment and bias

Based on the modified quality assessment criteria, two studies [71, 73] were classified as high quality, with scores ranging from 9 to 10. Five studies [66–68, 72, 74] were categorized as fair quality, with scores ranging from 5 to 8. Two studies [69, 70] fell into the low-quality category, with scores ranging from 0 to 4.

### Effects of simultaneous interventions

#### Effects on motor performance

Four studies combining simultaneous resistance and cognitive training improved motor performance over passive CG [67–69, 74]. Jardim et al. [67] and Nishiguchi et al. [68], reported gains in lower limb strength, gait speed, and TUG, while Norouzi al. [74] improved balance and Salazar-González et al. [69] improved DT walking speed. In two studies with active controls, both groups (Intervention group and CG) showed similar improvements in strength and mobility [66, 70].

### Effects on cognitive performance

Four studies examined simultaneous cognitive and resistance training compared to passive CG [67–69, 74]. Jardim et al. [67] used reaction time tasks, while the others combined resistance training with WM tasks (see Table 3). None of the studies showed effects on global cognitive performance.

Nishiguchi et al. [68] found improved simple reaction times, but similar gains occurred in the CG. Jardim et al. [67] reported enhanced attentional control on the Rapid Visual Processing (RVP) test and improved scores on the Paired Associates Learning (PAL) test and Consortium to Establish a Registry for Alzheimer’s Disease (CERAD) word list (evocation and recognition) for the intervention group only.

All four studies improved aspects of EF. Nishiguchi et al. [68] reported gains in the Trail Making Test (TMT (A-B)) and Wechsler Memory Scale Revised (WMS-R). Norouzi et al. [74] found improvements in n-back performance in both intervention groups (motor-motor DT and cognitive-motor DT). Salazar-González et al. [69] observed motor improvements in DT walking, but no cognitive effects.

Two additional studies [66, 70] used active CG. Laatar et al. [66] compared CME to balance/resistance training without cognitive tasks. Both groups improved processing speed, but CME showed more DT benefits. Uemura et al. [70] compared cognitive-motor resistance training with vs. without cognitive progression. Differences emerged only in DT walking performance (see Table 3), not in other cognitive outcomes.

### Effects of sequential interventions

Three studies using sequential training also reported mobility and strength gains [71–73]. However, Desjardins-Crépeau et al. [71] found handgrip strength increased only in active controls, with all groups improving in the Chair Rise Test. Across studies, gait speed, walking distance, and DT performance improved in both intervention and CG [71, 73]. Functional mobility also improved (Physical Performance Test (PPT) [71]; TUG [73]), with Van het Reve et al. [73] showing step length gains under DT conditions in the intervention group.

### Effects on cognitive performance

Three studies examined sequential cognitive and resistance training compared to active CG [71, 73].

Desjardins-Crépeau et al. [71] included four groups: (1) a main intervention group with visual discrimination DT and resistance, (2) a cognitive CG (computer-based tasks), (3) a cardio-strength group, and (4) a stretching group. All groups improved in attentional control, Color Word Interference Test (CWIT) inhibition, and TMT (A) However, only the main group improved on CWIT switching, and only the cognitive group improved on TMT (B) No group showed changes in Rey Auditory Verbal Learning Test (RAVLT).

Sipilä et al. [72] compared: (1) a main group with WM updating and resistance/home training, and (2) a combined machine strength, balance, walking, and aerobic training program. Both groups improved on the Stroop test (greater gains in the intervention group), TMT delta (A-B), and the CERAD test.

Van het Reve et al. [73] compared resistance training with virtual reality driving simulations to a CG doing strength and balance training. Both improved in processing speed (reaction time) and TMT (A-B), while only the intervention group improved in attentional control (Vienna Test System).

### Results according to F.I.T.T-V.P. principles ((frequency, intensity, type, volume and progression)

The outcomes of nine studies were analyzed using the F.I.T.T.-V.P. framework (frequency, intensity, time, and type plus volume and progression to assess combined cognitive and resistance training effects. Training frequency ranged from one to four sessions per week, with two to three sessions proving most effective (e.g [67, 68]). , , leading to improvements in gait speed (6-Minute Walking Test (6-MWT), 10-Meter Walking Test (10-MWT)), lower limb strength (STS), and functional mobility (TUG). Two weekly sessions also enhanced EF and attentional control [67, 71, 72], while processing speed improved with three cognitive and two strength sessions per week [73]. Memory gains were reported across interventions with one to three sessions weekly [66, 68, 74]. Training volume ranged from 150 min [70] to 6240 min [72], with 150 to 5200 min of cognitive training and 180 to 6240 of resistance training. Gains in gait speed were achieved with 180–720 min of training [66, 68, 71] whereas 720 min resulted in improvements in SPPB which includes gait, TMT and balance [73]. A volume of 360 min training enhanced processing speed and EF [73]. Memory improved after 1800 min [67].

Training intensity and progression varied: some studies used 60–70% max heart rate [67] or followed American College of Sports Medicine (ACSM) guidelines with biweekly progression (e.g [72]). , , applying progressive overload via increased load, repetitions, or task complexity [67, 68, 71]. All three studies reported improvements in at least one motor domain. In five studies, intensity and progression were not reported [66, 69–71, 74].

For progression in cognitive training, intensity was gradually increased in three studies [67, 68, 70], while two others applied automatic, performance-based adjustments [72, 73], such as tailored task speed. These approaches led to gains in EF, TMT (A-B), attentional control (Vienna Test System), and processing speed (reaction time).

Training dose also varied depending on the interventions content. Simultaneous interventions ranged from 150 min of cognitive training with < 600 min of motor training [70], to 1,800 min cognitive with > 720 min motor training [67]. Sequential interventions showed different dose requirements compared to simultaneous ones.

**Fig. 2:**
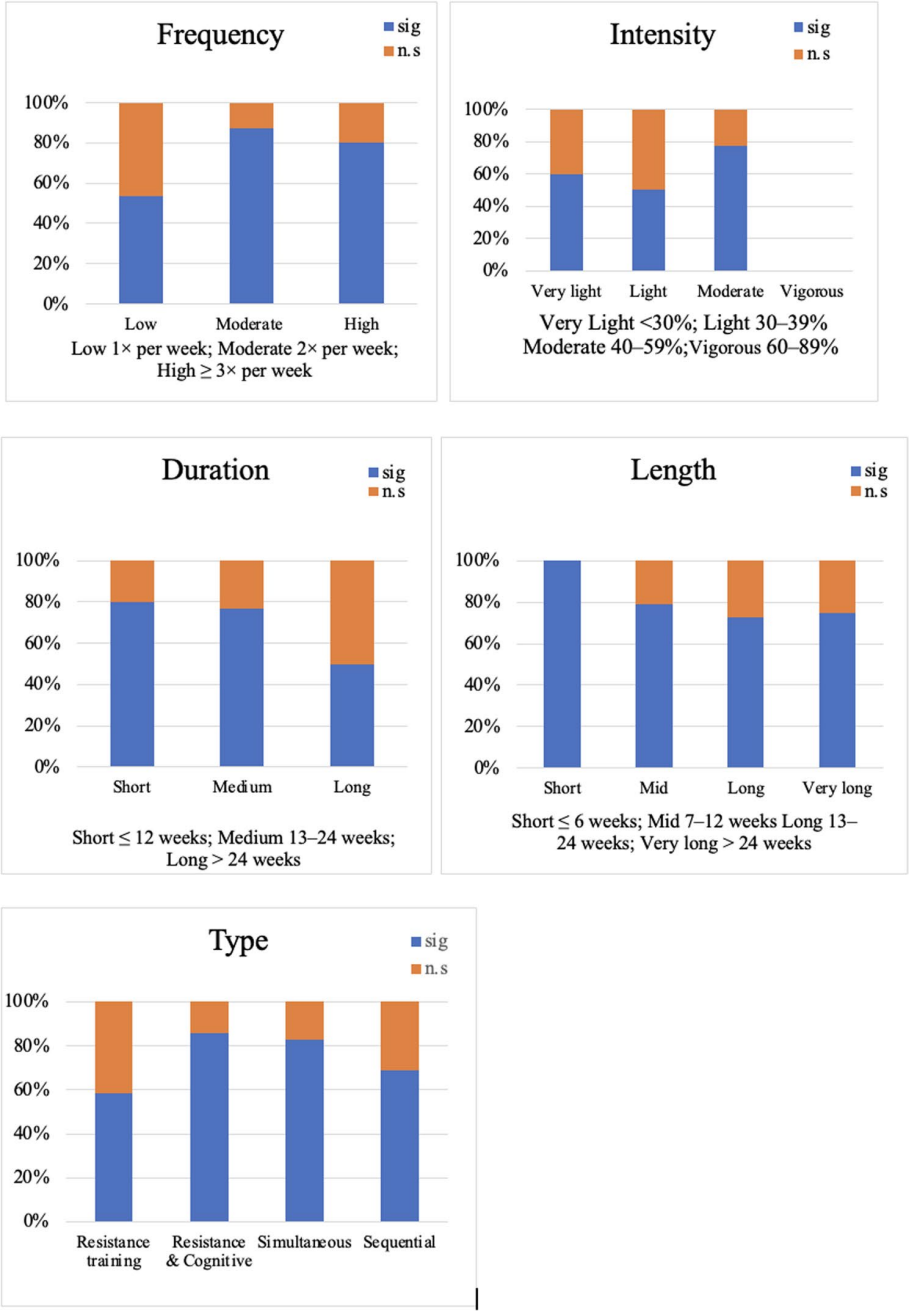
Number of studies reporting significant and non-significant effects of resistance training combined with cognitive training on motor and cognitive performance

As presented in the different parts of Fig. 2, higher frequencies, very light or moderate intensities, short or medium duration as well as a simultaneous conduction of cognitive and strength or resistance training showed the most significant changes of the outcomes.

## Discussion

This systematic review aimed to evaluate the effectiveness of resistance exercise combined with cognitive training to enhance muscle strength, mobility, and cognitive performance in individuals aged 65 years and older. The review had a specific focus on identifying the optimal combination of resistance and cognitive training, including training load parameters such as volume, frequency, intensity, and duration, and provide practical recommendations based on the findings.

### Effects of resistance training combined with cognitive training

Recent findings indicate that CME, which incorporates both motor and cognitive demands, is superior to traditional resistance training in improving DT-gait speed, balance, coordination, and real-life gait adaptability [30, 75, 76]. These complex interventions enhance sensorimotor integration and promote neuroplasticity, thereby contributing to improved postural stability in older adults [77]. CME, therefore, may support independence and reduce the likelihood of falls in this population [78].

In line with these previous studies five of nine studies measured muscle strength with four studies reported strength gains, mainly in lower limbs [66–68, 71]. Benefits were comparable to classical resistance training [24, 78], although high-intensity resistance training is the gold standard for strength improvement [79], our findings suggest that combining moderate- to high-intensity training with cognitive tasks can produce comparable gains in the target group of older adults [71]. Some interventions also improved balance [66, 80], supporting evidence for cognitive-motor integration [81]. Gait speed was improved regardless of a sequential or simultaneous design (e.g [66–68, 71, 72]). , . Functional mobility (TUG/Expanded Timed Up and Go (ETGUG)) improved in most studies [66, 67, 72, 73], often under DT conditions, indicating combined resistance and cognitive training benefits. Still, Desjardins-Crépeau et al. [71] and Nishiguchi et al. [68] found no TUG change, likely due to high baseline function, though even small gains may be meaningful for less robust populations [63]. While lower limb strength improved [66–68, 71], handgrip strength showed no change [71, 72] likely due to training dose or ceiling effects [82, 83].

Six studies found improved gait speed under DT conditions [66, 67, 69, 70, 72, 73], with both training types equally effective. These findings highlight cognitive-resistance synergy [30, 84] and support evidence favoring combined over traditional training [85, 86]. Van het Reve et al. [73] also reported reduced DT walking cost, with gains in step length and variability, key fall risk markers in older adults. Since gait slowing and shorter steps are linked to brain health [87] and fallers often show greater variability [88], CME may reduce fall risk by strengthening cognitive-motor integration [18, 89, 90].

Regarding cognitive performance, processing speed improved in three studies [66, 68, 73], with Jardim et al. [67] linking visual-auditory tasks to faster reaction times, though some CG gains suggest learning effects. Attention also improved consistently [67, 71, 73], particularly when complex cognitive tasks were embedded in resistance training [24, 91], with CME protocols outperforming cognitive training alone [91]. The reviewed studies show that incorporating progressively challenging cognitive tasks into resistance training enhances attentional control by increasing cognitive demands [73]. EF improved in six studies, including cognitive flexibility and set-shifting [68], WM, and inhibition [67], likely reflecting the combined effects of resistance exercise on cerebral perfusion and oxygenation [92–94] alongside cognitive activation of executive networks [36, 95, 96].

These positive results suggest that cognitive elements, especially attention and WM, may drive neural adaptations enhancing coordination and muscle activation [36, 80, 97]. This supports the assumption that engaging in cognitive stimulation alongside physical training, whether delivered simultaneously or sequentially, can enhance neuroplasticity, which is especially beneficial in mitigating age-related cognitive and physical decline [36, 80].

### Most effective training characteristics

Regarding the effects of combined training interventions, the results presented in the results section and illustrated in Fig. 2 reveal considerable heterogeneity. However, a consistent pattern emerges: interventions that were both structured and progressive, in terms of increasing physical intensity, cognitive challenge, or task complexity, tended to yield the most robust improvements in both cognitive and motor domains. For example, Jardim et al. [67] paired twice-weekly resistance sessions (including functional movements like squats and bench presses) with multisensory cognitive tasks over 12 weeks, is leading to a notable improvement in gait, limb strength, and multiple memory domains. Similarly, Sipilä et al. [72] implemented a year-long program combining resistance training with adaptive cognitive exercises and found significant gains in EF, gait speed, and DT performance. As shown in Fig. 2, the highest proportion of significant motor and cognitive improvements was reported in interventions with moderate to high frequency (≥ 2×/week), moderate to vigorous intensity, and durations spanning 13–24 weeks or longer. Notably, simultaneous training interventions, where resistance and cognitive tasks are performed within the same session, appeared to achieve cognitive and motor improvements with lower total training time, suggesting a greater efficiency. In contrast, sequential approaches required longer cumulative durations to reach similar effects. These findings align with neuroplasticity models, emphasizing that engaging cognitive and neuromuscular systems together may produce synergistic benefits that extend beyond those achieved through traditional training approaches [98].

Missing significant effects by Uemura et al. [70] might have been a result of low training dose (once weekly, < 25 min per session), which were considerably lower than in previous studies that implemented 45-minute sessions three times per week. This highlights the importance of training volume and progression in eliciting mobility improvements [44, 99]. Likewise, Sipilä et al. [72] found that both the intervention and active CGs showed improvements. However, the intervention group, which received fewer exercise components, reported gait gains similar to those of the CG, which engaged in more extensive multicomponent training, suggesting that multicomponent exercise alone may be sufficient.

In summary, the most effective combinations were those that were implemented simultaneously, progressively challenging, and perhaps most importantly, engaging for older adults. These findings underscore the importance of personalization and progression over standardized protocols. The key appears to be not just combining the two modalities, but designing them to complement and reinforce one another.

For the effects of the simultaneous cognitive-resistance training interventions we would like to introduce the picture of a “push-pull” effect. Regarding the components of submaximal strength/resistance training, muscle activity has a strength endurance component related to a higher cardio-vascular output and increasing blood flow. This increases the cerebral blood flow and accompanying metabolic effects, and in turn improves EF like WM. The pull effect results out of the initiation of cognitive processes resulting in higher attentional load and required cognitive resources. Regarding, for example, previous research by Herold et al. [36] or Tait et al. [37] for the mobility outcomes and combined cognitive motor performance, the simultaneous “push-pull” effect might be higher than some overlaps resulting from sequential interventions. Nevertheless, this idea needs to be proven in further RCT with equal training times for the cognitive and motor components.

### Recommendations for training load (frequency, intensity, type, volume and progression)

Findings underscore the importance of training frequency, intensity, volume, and progression, although outcomes varied depending on program configuration [1]. Interventions scheduling two to three sessions per week, lasting at least 30 min and sustained over 12 weeks or more, consistently led to performance improvements (Fig. 2).

Moderate to high intensity, particularly in resistance training, proved most effective. Following ACSM guidelines, Jardim et al. [67] incorporated training load progression by progressively increasing resistance loads or repetitions every two weeks, while Sipilä et al. [72] adjusted the intensity based on the participants performance, both applying progressive overload. A training volume of 720 min resulted in improved mobility, functional mobility and lower limb strength for the biweekly progression [67]. Individual progression improved mobility and DT-performance but did not improve lower limb strength at > 3000 min training volume [72], suggesting that strength improvements can be more successfully achieved when applying the ACSM recommendations. Gradually progression of cognitive training [67, 68, 70] and performance-based adjustments [72, 73] both resulted in improvements in the addressed domains. Only the study of Uemura et al. [70] is an example for lacking improvements despite progression most likely due to less overall time spent with training.

Moreover, programs combining resistance and cognitive exercises, especially when delivered simultaneously, achieved the highest proportion of significant outcomes (> 80%) (Fig. 2), highlighting the value of integrated approaches for enhancing cognitive-motor coupling and real-world functioning.

Volume was a key determinant of effectiveness. Interventions totaling ≥ 720 min of resistance training and a comparable amount of cognitive training produced the strongest effects. While 180–720 min were often sufficient to improve gait or mobility, cognitive outcomes, particularly in EF and memory, typically required > 2000 min. Improvements in the SPPB (indicating a gain in physical performance) were demonstrated after 720 min of sequential training by van het Reve et al. [73], whereas in Siplä et al. [72], the intervention did not lead to improvements despite a higher training volume (> 2000 min). A volume of 720 min of simultaneous training [68] led to significantly improved lower limb strength, which was not achieved when the intervention was delivered sequentially [72]. Notably, simultaneous training achieved comparable cognitive effects with approximately 720 min. In most cases, 600–720 min of cognitive training were sufficient for meaningful improvements [71]. (Fig. 2).

### Limitations

This systematic review has limitations that may impact the generalizability and consistency of its conclusions. Firstly, despite comprehensive inclusion of studies on combined resistance and cognitive training, the Cochrane Collaboration tool highlights concerns, including unclear specification of training components and insufficient information on intensity and progression. Limited detail on how training was adapted over time, especially in cognitive and motor aspects also challenges evaluation and affects generalizability of results, as reflected in the quality scores. This raises concerns about the therapeutic validity of several interventions reviewed, similarly as reported for the field of orthopedics [100, 101]. Publication bias may further constrain the review, as studies with significant outcomes are more likely published. Notably, all interventions combined resistance within the physical exercise programs, with unclear proportions and volume in some cases, raising questions about their specific impact. These issues underscore the need for cautious interpretation and suggest directions for higher therapeutic validity through clearer and standardized intervention reporting future research [102].

## Conclusion

This review shows that combining resistance and cognitive training can improve strength, gait, balance, processing speed, attention, and EF in older adults. Simultaneous training at moderate–high intensity, 2–3 times weekly for at least 30 min, appears most effective. Outcomes, however, vary with task complexity, intervention structure, frequency, intensity, and participant profiles, and not all studies found cognitive benefits. Heterogeneity in methods limits generalization, highlighting the need for rigorous long-term trials. Overall, integrated CME holds promise for reducing fall risk, maintaining independence, and enhancing quality of life, with future work needed to refine tailored, scalable protocols.

## Supplementary Information

Supplementary Material 1.

## Data Availability

No additional datasets were generated or analysed during the current study.

## Abbreviations

10-MWT: 10-Meter Walking Test
6-MWT: 6-Minute Walking Test
ACSM: American College of Sports Medicine
CCT: Controlled Clinical Trial
CERAD: Consortium to Establish a Registry for Alzheimer’s Disease
CG: Control Group
CME: Cognitive-Motor Exercise
CWIT: Color Word Interference Test
DT: Dual Task
EF: Executive Function
ETGUG: Expanded Timed Up and Go
F.I.T.T.V.P: Frequency, Intensity, Time, Type, Volume, Progression
PAL: Paired Associates Learning
PPT: Physical Performance Test
PRISMA: Preferred Reporting Items for Systematic Reviews and Meta-Analyses
PROSPERO: International Prospective Register of Systematic Reviews
RAVLT: Rey Auditory Verbal Learning Test
RCT: Randomized Controlled Trial
RVP: Rapid Visual Processing
STS: Sit to Stand
TMT: Trail Making Test
TUG: Timed Up and Go
WM: Working Memory
WMS-R: Wechsler Memory Scale Revised
