# Supplementary material for: Effects of different training characteristics in combined resistance and cognitive training on motor and cognitive performance in older adults: A systematic review
Source: Eur Rev Aging Phys Act. 2026 Jan 26;23:4. doi: 10.1186/s11556-026-00403-3 (PMC12849630; doi:10.1186/s11556-026-00403-3)
Supplement: Supplementary file 1 — Supplementary Material 1. [file 11556_2026_403_MOESM1_ESM.docx]

Table 4. Search overview systematic review

| Items | Details | Medline | APA PsycInfo |
| --- | --- | --- | --- |
| Keywords | 1 ("Age" or "elder" or "aged" or "senior" or "geriatric" or "eldest" or "aging" or "older adults" or "advanced age" or "geronic") | 7083120 | 1152546 |
|  | 2 ("resistance exercise" or "cognitive training" or "combined resistance and cognitive training" or "resistance training" or "cognitive training" or "practice" or "treatment" or "intervention of strength training" or "power training"). | 4663032 | 1097599 |
|  | 3 ("controlled" or "active control group" or "control group" or "inactive control group") | 1367177 | 215629 |
|  | 4 ("muscle strength" or "mobility" or "motor" or "cognitive function" or "cognitive performance" or "cognition" or "dual task" or "coupled task" or "secondary task" or "strength") | 886979 | 492809 |
|  | 5 (“weight training” or free-weights” or “weight machines”) | 4776 | 1236 |
| Filters | 6 limit 5 to (abstracts and english language and male and female and humans and yr="1994 -2025" and "all aged (65 and over)" and journal article),7 limit elderly - focussed | 1993 | 2525 |

Table 5. Search overview systematic review

| Items | Details | Web of Science |
| --- | --- | --- |
|  | (AB=("cognitive training") OR AB=("cognitive exercise") OR AB=("combined cognitive"  training) OR AB=("cognitive training intervention") OR AB=("cognitive performance")) AND  (TI=(cog*) OR (TI=(train*)) | 32368 |
|  | AB=(exercise) OR AB=("resistance training") OR AB=(practice)OR AB=("power  training") OR AB=("strength training") OR AB=("motor training") | **4,174,048** |
|  | elder (All Fields) OR geriatric (All Fields) OR eldest (All Fields) OR aging (All Fields)  OR older adults (All Fields) OR advanced age (All Fields) OR geronic (All Fields) OR Age (All  Fields) OR aged (All Fields) OR senior (All Fields) | **11,615,875** |
|  | muscle strength (All Fields) OR mobility (Abstract) OR motor (Abstract) OR cognitive  function (Abstract) OR cognitive performance (Abstract) OR dual task (Abstract) OR coupled  task (Abstract) OR secondary task (Abstract) OR strength (Abstract) | 9,915,071 |
|  | weight training (All Field) OR free-weights (All Field) OR weight machines (All Field) | 191 |
| Filters | #1 AND #2 AND #3 AND #4 AND #5 | 702 |
|  | #6 NOT#7 and Article (Document Types) and English (Languages) and Article  (Document Types) | 288 |
| Records assessed for eligibility from three data bases (Medline, APA PsycInfo, Web of science) |  | 33 |
| Studies included in review  from three data bases(Medline, APA PsycInfo, Web of science) |  | 9 |
